# Orthodontic treatment with passive eruption and mesialization of semi-impacted mandibular third molar in an adult with multiple dental losses

**DOI:** 10.1590/2177-6709.24.6.036-047.oar

**Published:** 2019

**Authors:** Armando Yukio Saga, Ariane Ximenes Graciano Parra, Isteicy Cortêz Silva, Cayana Dória, Elisa Souza Camargo

**Affiliations:** 1 Pontifícia Universidade Católica do Paraná, Escola de Ciências da Vida (Curitiba/PR, Brazil).; 2 Pontifícia Universidade Católica do Paraná, Escola de Ciências da Vida, Programa de Pós-Graduação em Odontologia (Curitiba/PR, Brazil).; 3 Pontifícia Universidade Católica do Paraná, Escola de Ciências da Vida, Graduação em Odontologia (Curitiba/PR, Brazil).

**Keywords:** Orthodontics, Tooth movement, Angle Class II malocclusion

## Abstract

**Objective:**

This article describes the orthodontic treatment performed on an adult patient with multiple dental losses.

**Case report:**

A female patient, 20 years and 4 months old, presented with the following conditions: absence of teeth #26, #35, #36 and #46; semi-impacted tooth #48; inclined molars adjacent to an edentulous space; canines and premolars in a Class II relationship; a convex profile; biprotrusion; and forced lip sealing.

**Results:**

Space in the region of tooth #26 was closed, as well the space of tooth #46; tooth #48 erupted and followed mesial movement passively; space of the region of tooth #35 was maintained for the placement of a dental implant; uprighting of tooth #37 was obtained. Aesthetic and functional goals of the treatment were achieved. Results remained stable 10 years after the end of the treatment.

**Conclusion:**

The modified helical loop could be effectively used in orthodontic mechanics to close edentulous spaces. Passive semi-impacted mandibular third molar eruption and mesialization can occur in adults when proper space is provided.

## RESUMO

**Objetivo:**

o presente artigo descreve o tratamento ortodôntico realizado em uma paciente adulta que apresentava múltiplas perdas dentárias.

**Relato do caso:**

paciente do sexo feminino com 20 anos e 4 meses de idade, apresentava as seguintes condições: ausência dos dentes #26, #35, #36 e #46; dente #48 semi-impactado; molares adjacentes ao espaço edentado inclinados; relação Classe II de caninos e pré-molares; perfil convexo; biprotrusão; selamento labial forçado.

**Resultados:**

foram obtidos o fechamento do espaço da região do dente #26, assim como do #46; o dente #48 irrompeu e acompanhou esse movimento mesial; manutenção do espaço da região do dente #35 para colocação de implante dentário; e verticalização do dente #37. Os objetivos estéticos e funcionais do tratamento foram alcançados. Os resultados alcançados permaneceram estáveis 10 anos após o fim do tratamento.

**Conclusão:**

a alça helicoidal modificada pode ser efetivamente usada na mecânica ortodôntica para fechar espaços edêntulos. A erupção passiva e a mesialização de terceiro molar mandibular semi-impactado podem ocorrer em adultos, quando espaço adequado é obtido.

## INTRODUCTION

Orthodontic treatment is increasingly being pursued by adult patients and it could require a multidisciplinary approach. Orthodontic treatment has been performed on many adult patients who have suffered tooth loss and prolonged absence of teeth, conditions that may limit treatment.^1^ Increases in life expectancy, quality of life, and aesthetic requirements have led to increases in the number of adults interested in orthodontic treatment.^2^ ^,^ ^3^ The improved comfort and aesthetics of orthodontic appliances have also encouraged adults.^4^ ^,^ ^5^

The adult patient may have certain conditions such as edentulous areas, abnormal tooth inclinations, and periodontal infections.^6^ ^,^ ^7^ Premature loss of posterior teeth, usually the first molars, is common.^8^ A delay in the replacement of a lost tooth can cause inclinations of the adjacent teeth, extrusion of the antagonist, increase in the overbite, temporomandibular joint dysfunctions, soft tissue disorders, bone loss, and occlusal interferences; these abnormalities can hinder a possible prosthetic rehabilitation.^9^

Since the tooth adjacent to an edentulous space tilts, the gingival tissue is modified. This results in a periodontal pocket that prevents proper oral hygiene and leads to bacterial plaque accumulation at the site, which may cause periodontal tissue injuries.^10^ Such consequences may be aggravated by misdirected forces resulting from dental inclination.^11^ In order to preserve the integrity of occlusion, teeth and tissues adjacent to the tooth loss, the treatment plan can either include orthodontic closure of the space, maintaining or opening the space for prosthetic rehabilitation.

In this paper, an orthodontic treatment of an adult patient with bilateral posterior tooth loss is reported. The treatment comprised three main procedures: 1) maxillary first pre-molar extraction and anterior teeth retraction; 2) space closure in the right mandibular side which bone structure allowed movement and uprighting; 3) light mesialization of the tooth adjacent to the edentulous space on the opposite side, for prosthetic rehabilitation in left mandibular side.

## DIAGNOSIS

**Figure 1:**
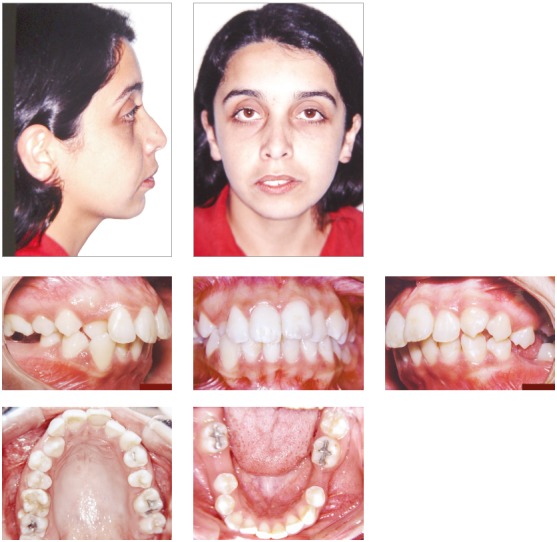
Initial facial and intraoral photographs.

The female patient, aged 20 years and 4 months, complained that the maxillary incisors were protruded and that dental losses had occurred. Her medical history showed no contraindication to orthodontic treatment. The extraoral examination revealed moderate facial asymmetry (left side larger than right one), that lead to an occlusal plane cant, absence of labial sealing, a convex profile, upper and lower lips well positioned, and an increased labiomental groove (Fig 1). 

**Figure 2:**
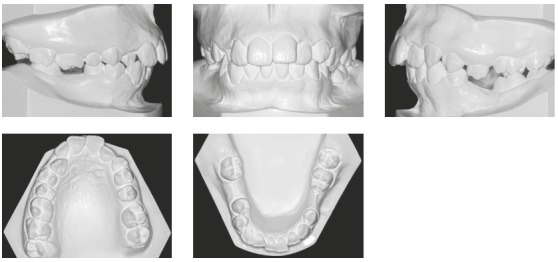
Initial dental casts.

She had no signs or symptoms of temporomandibular dysfunction. The intraoral analysis revealed dental midlines coinciding with each other and with the facial midline; analysis also revealed: 6-mm overjet, 5-mm overbite, discrepancy of -1 mm in the mandibular anterior region, protruding maxillary and mandibular incisors, crossbite at tooth #13, and the absence of teeth #26, #35, #36 and #46 (Figs 1 and 2).

The mandibular second and third molars on the left side (#37 and #38) were mesially inclined, and the left first premolar (#24) extruded because the mandibular left first molar (#36) and the mandibular left second premolar (#35) were absent. The mandibular right second molar (#47) was inclined mesially because the mandibular right first molar (#46) was absent. Class II canine and premolar relationships were observed bilaterally, and the curve of Spee was moderate.

**Figure 3:**
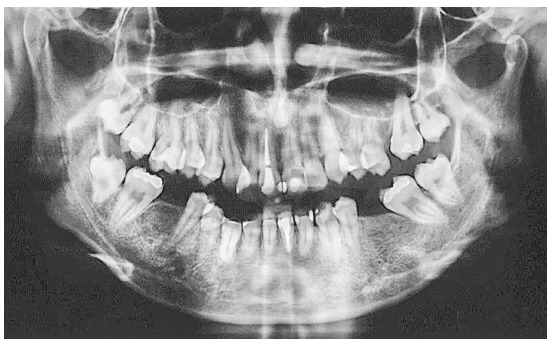
Initial panoramic radiograph.

Panoramic radiography showed absence of caries or pathologies (Fig 3). The maxillary right central incisor (#11) and the mandibular left central incisor (#31) were endodontically treated. The region of the mandibular left first molar (#36) had bone defect, and the mandibular right third molar (#48) was present but had not erupted and was semi-impacted.

**Figure 4:**
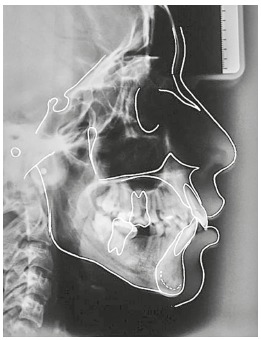
Initial cephalogram and cephalometric tracing.

The initial cephalogram and cephalometric tracing showed maxillary prognathism but good mandibular positioning (SNA = 85^o^ and SNB = 78.5^o^), results that confirmed a Class II skeletal pattern (ANB = 6,5^o^) and a dolichofacial facial form (SN-GoGn = 35.5^o^, FMA = 29^o^) (Fig 4). The maxillary incisors were lingually positioned and slightly protruded (1.NA = 15.5^o^ and 1-NA = 6 mm), and the mandibular incisors were vestibularized and protruded (1.NB = 31^o^ and 1-NB = 9 mm) with good interincisal angle (126.5^o^) (Table 1).

**Table 1:** Initial (A) and final (B) cephalometric measurements.

Measurements	Normal	A	B	Dif. A/B
SNA (degrees)	82	85	84	1
SNB (degrees)	80	78.5	77.5	1
ANB (degrees)	2	6.5	6.5	0
SN-GoGn (degrees)	32	35.5	32	3.5
1.NA (degrees)	22	15.5	5.5	10
1-NA (mm)	4	6	1	5
1.NB (degrees)	25	31	27	4
1-NB (mm)	4	9	7	2
Interincisal angle (degrees)	131	126.5	141	14.5
Pog-NB (mm)	-	0	1	1
Upper lip - S-line (mm)	0	2.5	1	1.5
Lower lip - S-line (mm)	0	3.5	1.5	2
FMA (degrees)	25	29	26.5	2.5
FMIA (degrees)	65	55.5	58.5	3
IMPA (degrees)	90	95.5	95	0.5
Z-Angle (degrees)	75	63	68.5	5.5

## TREATMENT ALTERNATIVES

An option was to perform an orthognathic surgery. However, because patient did not want to undergo a major operation, she rejected this treatment option. Alternatively, orthodontic treatment could comprise extraction of mandibular third molars and uprighting of the second molars to prepare spaces to dental implants. But the patient wanted to reduce the number of prosthesis. 

## TREATMENT OBJECTIVES

1) Extract maxillary first premolars (#14 and #24) to retract anterior teeth and: position the canines in Class I relationship; decrease overjet and obtain canine’s and incisor’s guide; reduce labial biprotrusion; achieve passive lip seal and improve facial profile.
2) Improve smile aesthetics by correcting the crossbite at the maxillary right canine (#13) and aligning and leveling the maxillary and mandibular arches.
3) Correct maxillary and mandibular dental crowding.
4) Maintain the space for rehabilitation with a dental implant in the region of the mandibular left second premolar (#35) and upright the mandibular left second molar (#37).
5) Close the space resulting from the loss of the mandibular right first molar (#46) and upright the mandibular right second molar (#47). Patient was aware that if the right third molar was ankylosed, a dental implant would be necessary distal to the second molar.
6) Obtain a normal overbite by intrusion of the maxillary incisors.

## PROGRESS OF TREATMENT

Initially, the maxillary and mandibular fixed appliances were installed with 0.022-in standard Edgewise brackets; the patient was then referred for the extraction of the maxillary first premolars (#14 and #24). The sequence of archwires used for aligning and leveling the teeth was as follows: 0.016-in NiTi, 0.016-in stainless steel, 0.018-in stainless steel, and 0.020-in stainless steel.

The maxillary extraction spaces were closed with a stainless steel, rectangular, 0.018 × 0.025-in retraction archwires with a loop distal to the canines.

**Figure 5:**
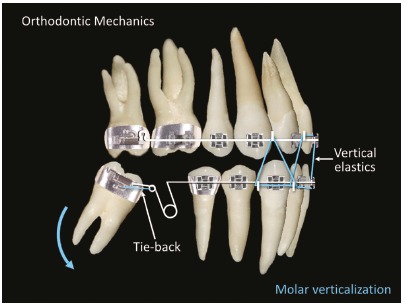
Orthodontic mechanics: mandibular second molar uprighting.

To upright the mandibular left second molar (#37), a helical open loop was used passively without any mesialization force. The molar was attached to the small helicoid present at the distal portion of the loop so that the force was applied at the tooth’s center of rotation. Space was maintained for future implantation in the edentulous region (Fig 5).

**Figure 6:**
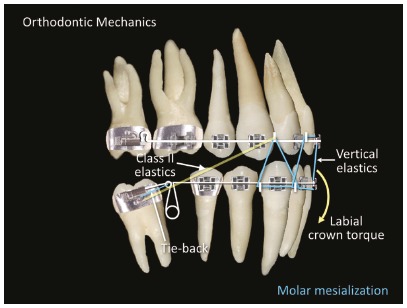
Orthodontic mechanics: mandibular second molar mesialization.

The open helical loop and the technique used for the mandibular left second molar (#37) was also used for the mandibular right second molar (#47) (Fig 5). Therefore, for tooth #47, the helical loop worked passively as an alignment and leveling loop. After the second molar was uprighted, the helical loop was activated to mesialize the molar and retract the anterior teeth. Effective tip-backs of 20^o^ to 30^o^ were applied to correct the mesial inclinations of the second molars. A slight toe-in was necessary to prevent their mesial rotations. To prevent excessive retraction of the mandibular anterior teeth, Class II elastics were used, and active vestibular torque was applied to the mandibular incisors (Fig 6).

No mini-implants or miniplates were employed. After the space in the region of the absent mandibular right first molar (#46) was closed, the mandibular right third molar (#48) passively erupted and followed the mesial movement and was included in the archwire afterwards.

To finish the treatment, a 0.019 x 0.025-in stainless steel archwire was used on each dental arch. When appliances were removed, a maxillary wraparound retainer was placed, and a mandibular lingual wire retainer was bonded from canine to canine. A 0.016-in stainless steel segments were used for three months to retain the mandibular second molars.

## TREATMENT RESULTS

**Figure 7:**
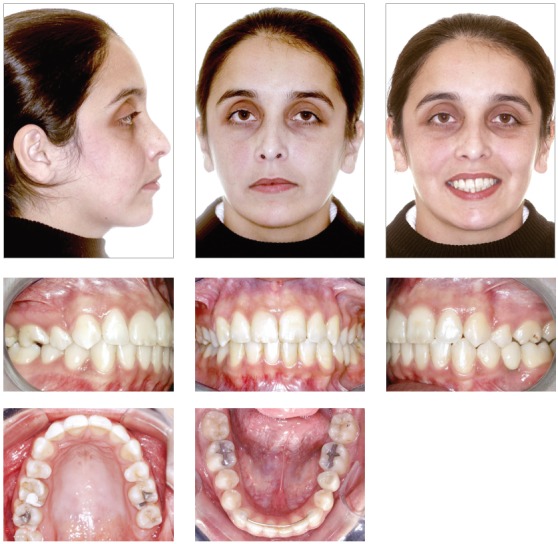
Final facial and intraoral photographs.

**Figure 8:**
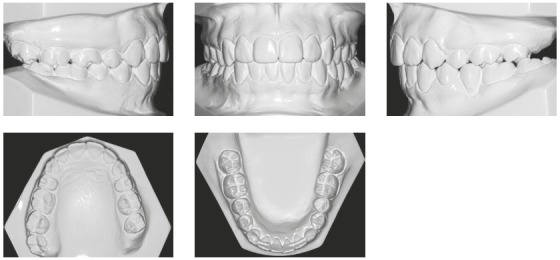
Final dental casts.

**Figure 9:**
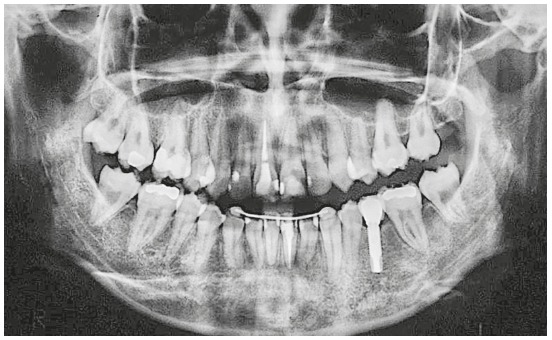
Final panoramic radiograph.

At the time of the post-treatment extraoral examination, the patient’s facial profile had improved, and her lip seal had no abnormal muscular contractions. When smiling, an improvement in aesthetics occurred caused by dental alignment and protrusion reduction, but there was still a cant in occlusal plane caused by facial asymmetry - which was expected (Fig 7). The intraoral examination revealed that dental alignment and leveling were obtained, and that tooth intercuspation was satisfactory. The premolars and canines were in Class I relationship. The inclination of the occlusal plane persisted at the end of the treatment, as expected by the mechanics employed (Figs 7 and 8). Overbite and canine’s crossbite were also corrected. In the panoramic radiograph, it was observed uprighting of the mandibular second molars (#37 and #47) and greater root movement than in the crown (Fig 9).

**Figure 10:**
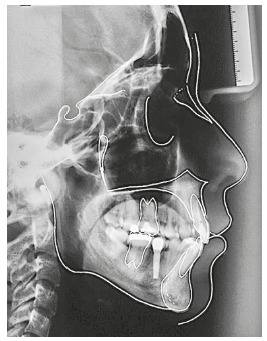
Final cephalogram and cephalometric tracing.

**Figure 11:**
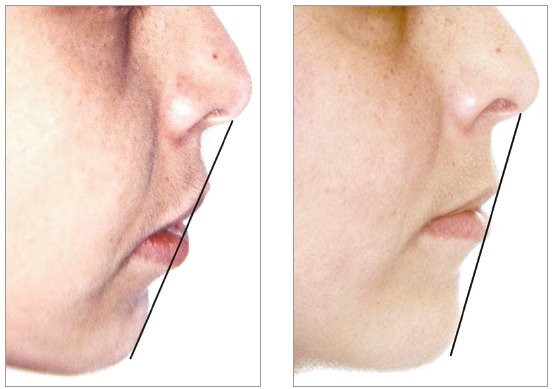
Initial and final S line.

Through cephalogram and cephalometric tracing (Fig 10), it was verified that the skeletal anteroposterior relationship (ANB) was maintained. The maxillary incisors were repositioned (from 15.5^o^ to 5.5^o^), which resulted in an improvement in the labial position with respect to the S line (from 2.5 mm to 1 mm at the upper lip and from 3.5 mm to 1.5 mm at the lower lip) and, thus, better lip sealing (Fig 11, Table 1). 

**Figure 12:**
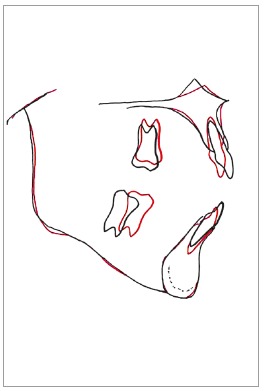
Superimpositions of initial (black) and final (red) cephalometric tracings.

**Figure 13:**
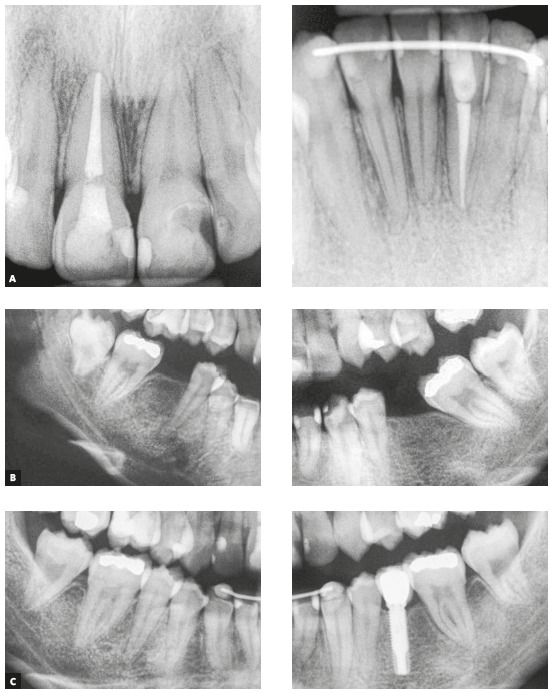
Final (A) periapical radiographs of maxillary and mandibular incisors, initial (B) periapical radiographs of the edentulous regions and final (C) periapical radiographs of the verticalized and mesialized mandibular second molars and the dental implant replacing tooth #35.

In the superimposition of the initial and final cephalometric images (Fig 12), the following can be observed: retraction and intrusion of the maxillary incisors, more accentuated intrusion and slight linguoversion of the mandibular incisors; intrusion of the maxillary right first molar (#16), and uprighting and mesialization of the mandibular right second molar (#47). As can be seen in the periapical radiographs, root resorption in the molars and incisors was minimal (Fig 13).

**Figure 14:**
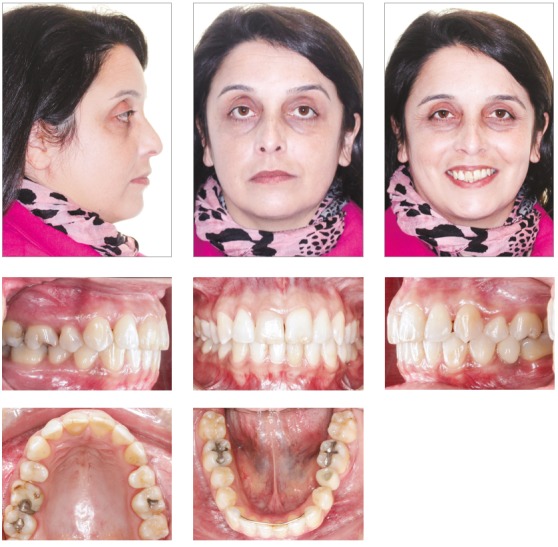
Facial and intraoral photographs of 10-years post-retention follow-up.

**Figure 15:**
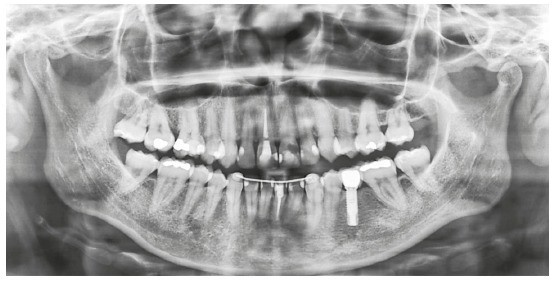
Panoramic radiograph of 10-years post-retention follow-up.

The treatment lasted 3 years and 4 months; the goals were achieved, and the patient was satisfied with the result. In the exams of the 10-years post-retention follow-up (Fig 14), the stability of the dental and facial corrections can be observed and maintenance of the teeth space closures as well (Fig 15).

## DISCUSSION

The three-dimensional control of dental movement during uprighting and the closure of spaces are of paramount importance for meeting treatment objectives.^12^ Because the molar roots are bulky, the movements become difficult to control and may cause undesired effects. To apply adequate force, the orthodontist must consider several factors, such as the presence or absence of other permanent teeth, the degree of mesial and/or lingual inclination of the molar, and the need for anchorage.^13^

It is important that space closure occurs without causing injury to supporting tissues. Therefore, it is desirable that the movement be performed without the formation of extensive areas of hyalinization, which may hinder and delay this movement.^14^ It is necessary that the applied force produce an effective movement with minimum discomfort and minimum damage to the tissues.

The acute angles formed between the inclined molars and the alveolar bone contribute to the formation of periodontal pockets and bone defects; thus, molar upright can improve the alveolar bone contour^15^. Uprighting minimizes or completely eliminates infrabony pockets because the alveolar bone could accompany the cementoenamel junction as the tooth is verticalized.^6^ In the presented case, the improvement of the periodontal pocket can be observed because of the molars’ uprighting.

For molars uprighting, light and continuous forces are recommended, as is the control of occlusal trauma to minimize root and bone resorption.^16^ Despite the careful application of the forces, panoramic post-treatment radiography revealed a slight rounding in the radicular apices of the anterior teeth, a finding commonly related to orthodontic treatment.^17^

As a response to the uprighting of an inclined mandibular molar, extrusion can occur and consequently lead to the opening of the bite in the anterior region. If extrusion is not desirable, the uprighting must occur with an intrusion movement or extrusion control.^9^ In this case, extrusion was controlled with vertical elastics in the anterior region.

The absent mandibular left second premolar (#35) was replaced with a dental implant with the intention of maintaining the symmetry of the arch. Other studies^18^ ^,^ ^19^ have shown long-term success with orthodontic movement and the placement of dental implants in edentulous spaces.

According to Zachrisson,^20^ the orthodontic movement of a tooth is an excellent method, perhaps the best and most predictable method, for regenerating the alveolar bone and adjacent tissues. The width of the alveolar bone can be modified by the orthodontic treatment because the bone accompanies the tooth as it moves to the edentate space.^15^ Hom and Turley^11^ found that to reach the greatest amount of space closure and the least amount of molar bone loss, the ideal size of the space of the first mandibular molar is 6 mm or less of mesiodistal length and 7 mm of vestibular-lingual thickness. Controlled anchoring is important in this type of movement, because the excessive linguoversion of the mandibular incisors should be prevented during the mesialization of the molar.^21^ In the presented case, linguoversion was controlled with buccal torque applied to the mandibular incisors.

Several movements were used to obtain differential anchorages.^12^ ^,^ ^22^ The helical loop was adequate for the closure of the space in the atrophic bone. The effects observed during this closure were acceptable; however, some vertical bone loss and gingival recession occurred at the second molar (#47). Despite these mild adverse effects, this tooth had no mobility or painful symptomatology.

Some teeth have a greater tendency to relapse after being moved. Therefore, a continuous retainer should be used to allow bone remodeling at the site and a stable dental position.^9^ In this case, in addition to the conventional appliances (maxillary wraparound and mandibular fixed lingual retainer), a 0.016-in stainless steel archwire was placed for the retention of the mandibular second molars for three months. Because efficient mechanics were applied and the retention devices were correctly used, the case had remained stable 10 years after the end of the treatment.

This case report shows that molar uprighting and closing the spaces of lost molars can be a viable solution. To evaluate the health of the dental roots and the surrounding alveolar bone, clinical examinations and periapical radiographs^1^ are crucial during the uprighting of molars. To verify the stability of space closures, these follow-up assessments are also critical during and after the period of retention.

## CONCLUSION

The reported case shows that orthodontic techniques, together with methods from other dental specialties, are able to adequately resolve the sequelae left by dental losses. Molar uprighting and space closure with modified helical loops are simple and efficient and, when correctly employed, allow dental movement to be precisely controlled. 
